# Vaccination against Hepatitis B: A Scoping Review

**DOI:** 10.31557/APJCP.2020.21.12.3453

**Published:** 2020-12

**Authors:** Parimala Mohanty, Pratap Jena, Lipilekha Patnaik

**Affiliations:** 1 *Department of Community Medicine, IMS & SUM Hospital, Siksha ‘O’ Anusandhan Deemed to be University, Bhubaneswar, India. *; 2 *School of Public Health, KIIT Deemed to be University, Bhubaneswar, India.*

**Keywords:** Hepatitis B, immunization, barriers, sustainable development goal

## Abstract

**Objective:**

Elimination of viral hepatitis by 2030 as one of the international Sustainable Development Goals puts the hepatitis B vaccination on the forefront. However, barriers to vaccination reported in various studies are of concern. This study explores the global barriers for effective uptake of Hepatitis-B vaccination.

**Methods:**

A scoping review of studies reporting hepatitis B vaccination barriers was done using PMC data base and Google scholar search engine. About 803 journal articles and reports on hepatitis B barriers were retrieved but only 36 most relevant items during last 10 years were identified, pile sorted, grouped and analyze.

**Results:**

Overall 74 barriers have been identified for effective uptake of hepatitis-B vaccines. Most studies focused on non-zero dose of hepatitis B vaccine, One-third of the barriers are related to system issues, one-fourth of the barriers were related to caregiver education or awareness, fear of side effect, migration etc., one-fifth barriers were related to service provider issues like poor out-reach, home visits, poor communication and/relation with the caregivers, failure to identify unimmunized children etc., and other barriers were social-cultural issues. The review reveals limited availability and accessibility to health-facility based immunization, lack of awareness among caregivers, poor communication by the healthcare workers and negative relationships with the beneficiaries, cost of vaccine in private sector, inconvenience time and place of vaccination etc. as the major barriers for hepatitis B vaccination. Barriers varied from country to country.

**Conclusion:**

Myriad barriers for reduced hepatitis-B vaccine uptake need to be addressed contextually as countries are at different stages of hepatitis-B vaccination implementation.

## Introduction

Hepatitis B viral (HBV) infection is a vaccine-preventable disease which puts an increased economic burden on families, communities, and the country. Around the globe HBV infection poses a major concern as it roots to top ten reason for mortality and is 50–100 times more infective than human immunodeficiency virus (Kew, 2012).

Humans are the only known natural host (Chingle et al., 2017) and transmission occurs by infection through various body fluids vertical, sexual activities, in addition infection may be caused by use of common syringes (Shepard et al., 2006a; Struve et al., 1990). Health Behavioral factors also varies across different ethnic groups (Maxwell et al., 2012).

Worldwide, more than 2 billion of the population has been infected with HBV and 50 crores individuals are likely to be affected by HBV (Shepard et al., 2006b). This virus kills 1.5 million people a year, and one in every three people have been exposed to the virus with most infected people being asymptomatic (*Hepatitis B - Vaccination of Adults| CDC, 2019*). More than 780,000 people die every year due to complications of HBV infection. During traveling to high endemic area with prevalence of more than 2% HBV vaccination is advocated. (*Hepatitis B - Vaccination of Adults | HBV | Division of Viral Hepatitis | CDC, 2019*). 

HBV causes acute and chronic hepatitis resulting in cancer and cirrhosis of liver. Treatments with antiviral agents have limited effect and so in order to safeguard from the disease preventive approach should be opted. To eradicate the infection universal vaccination is needed but is yet to be adopted by all countries. (Tajiri and Shimizu, 2015). 

Besides the direct health effects, HBV patients suffer from both discrimination (Yu et al, 2015) and health issues like mental illness and low quality of life (Fife and Wright, 2000). Fear and anxiety leading to social isolation and intensification of stigma related to HBV (Marinho and Barreira, 2013) occurs due to denial of job resulting in financial burden (Leng et al., 2016). 

There is no known cure as prevention is the only safe strategy against the high prevalence of viral hepatitis B. Safe and effective HBV vaccines have been available since 1982 (*WHO | Global Health Sector Strategy on Viral Hepatitis 2016-2021, n.d.*). The World Health Organization(WHO) has recommended the implementations of mass immunization programs since 1991 (Poland and Jacobson, 2004) and policy for all countries by the year 1995 having a HBV carrier rate of ≥8% to include vaccination into the national immunization programs (Komatsu, 2014). A recommendation to include birth dose within 24 hours of birth to all newborns by has been made for high efficacy and protectiveness (WHO Publication, 2010). 

The chance of developing chronic infection is more among the infants who get infected at birth, adults including sex workers, transgender, healthcare workers (HCWs), prison inmates, injection drug users, indigenous population, transplant patients, people living with HIV and pregnant women at risk. These groups are recommended for taking HB vaccination (WHO Publication, 2010).

Immunization uptake has been influenced by several barriers like less knowledge regarding HBV and its symptom, those who have no time to do test or don’t have a health insurance (Taylor et al., 2013), people attitude for vaccine and its awareness, how they perceive disease and health, social factors, habits etc. (Wheelock et al., 2013). Some of the barriers include misperceptions that vaccines are just for children prevails among general public and even among healthcare providers. Logistical issues related to vaccine delivery, inability to determine immunization status and funding for adult vaccines and vaccine visits also barriers to hamper vaccination coverage (Swanson et al., 2015). Several strategies have been formulated to achieve the immunization coverage within the adult group (Poland et al., 2010), but little has been achieved and the headway has been sluggish (MacDougall et al., 2015a).

We evaluated factors independently associated with barriers to hepatitis B vaccine and to identify the barriers against the immunization.

## Materials and Methods

*Search Method*

Research papers published are searched using these databases: Pub Med, Pub Med Central, and goggle scholar. Papers are identified and the following keywords were searched: “*Hepatitis B vaccine AND Barrier”, “Facilitators to Hepatitis B Vaccination*”, and, “*Barrier vaccination*”.

*Inclusion Criteria*

Those studies were included which were based on Studies barriers and facilitators of Hepatitis B vaccination and written in English.

*Exclusion Criteria*

Those studies were not included which was based on evidence reports, comparative studies, review paper and taken down in other language.

*Search Outcome*

From the search 803 studies were extracted, which was written in English. Those excluded are on the following basis:

Exclusively information on disease specific and do not help in generalization-306, No direct reference to barriers-188, clinical guidelines and no information on barrier-249.

**Figure 1:**
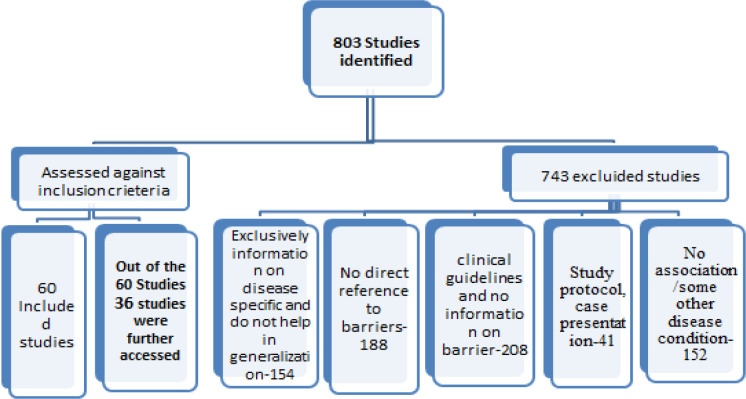
Outline of Search Strategy

Rest 60 studies further were evaluated seen whether it fulfilled the objective. To begin with first the papers were selected by overlooking the abstract, then the full papers were examined if it met the inclusion criteria.743 studies, did not meet the inclusion criteria, and so were excluded from the review. The process of selecting the final 60 studies is outlined in Figure 1.

*Data Abstraction*

Further out of these 60 studies, 36 studies were included as it fulfilled the objective of the study. Barriers of Hepatitis B vaccine were identified and extracted from these studies. The survey participants were asked to choose barriers that are pertinent to them and group barriers together by using Pile sorting/card sorting technique. This is a robust method by using which relationship between individual and group norms can be reconnoitered. We instructed the participants to segregate the cards into different groups as per their insight to various barriers. The participants are instructed to group cards based on their perceptions of the barriers. Sequential to this, cards with barrier mentioned in it having homogeneity or closeness were grouped into one category.

The process creates unconstrained clusters and continued until all cards were grouped into pile. Total four piles were formed named “*System Issue*”, “*User Issue*”, “*Service Provider Issue*” and “*Socio-Culture Factor*”. The barriers were listed into each group and analysis is done.

## Results

*Characteristic features of the Studies*

Out of 36 studies 21 studies have been conducted by cross sectional survey design, 4 studies have used qualitative study design and only 2 studies have been conducted by retrospective design, 1 case control, 1 randomized controlled trial and 1 mixed methodology. 

The size of sample varied and stretched from 6 to 18,046, retrospective study had the lowest sample size among all studies. Samples found in the reviewed studies had great variation among different age and working sections (infant, adolescence, adults, nurse, patients, pregnant women, GP, doctors, health professionals, immigrants, STD clinics, injection users) in hospital and community settings Only 8.3% (n=3) of the studies are conducted in African countries, 19.4 % (n=7) from Asian countries and rest 72 % (n-26) studies are conducted by developed countries. 

*Barriers to Utilization of Hepatitis B Vaccine*

Overall, seventy-four barriers have been identified which affect the effective expand of hepatitis-B vaccines uptake. These barriers are not similar in nature and varies across countries, different target group, 55% (n=20) of study are among low risk general population. Most studies focused on non-zero dose of hepatitis B vaccine. According to the review, the result highlights that the 36 studies linked to barriers underlines mostly on lack of awareness on HBV in general population and high risk group, caregiver education, cultural beliefs, fear of side effect, parent concern on adverse effect, lack of awareness, vaccine confidence gap, health worker poor knowledge about dose administration, insufficient screening of high risk group, poor communication, insufficient training of health workers, long delays to vaccine access, high costs of HBIG, cold chain issue etc.

“*Lack of knowledge about Hepatitis B vaccination and having low education among care giver*” is mentioned in more than 33% (12) of the studies. Followed by “*False belief regarding vaccination and cultural barriers*” also mentioned by 25% (9) of studies and similarly “*Cost of vaccine and unavailability of the vaccine*” mentioned by 25% (9) of reviewed studies. The fourth most mentioned barrier is “*low awareness among providers and Inadequate knowledge of HBV among health-care workers*” which mentioned by 19% (7) of reviewed studies. While other barriers such “*Time constraints by care giver*”, “*language barrier*” “*lack of funding for adult vaccines*” ,“*logistical issues related to vaccine delivery” ,“immigrant women*”, “*ethnicity and place of origin being a barrier*”, “*Insurance was a barrier in a minority*” was mentioned in few studies.

The identified 74 barriers according to the perceptions of the participants the cards with barriers names are sorted into “*System Issue*”, “*User Issue*”, “*Service Provider Issue*” and “*Socio-Culture Factor*”. One-third of the barriers are related to system issues, one-fourth of the barriers were related to caregiver education or awareness, fear of side effect, migration communication and/relation with the caregivers, failure to identify unimmunized children etc., and other barriers were social-cultural issues. The review reveals limited availability and accessibility to health-facility based immunization, lack of awareness among caregivers, poor communication by the healthcare workers and negative relationships with the beneficiaries, cost of vaccine in private sector, inconvenience time and place of vaccination etc. as the major barriers for vaccine uptake. Barriers varied from country to country. etc., one-fifth barriers were related to service provider issues like poor out-reach, home visits, poverty (Table 1).

**Table 1:** Pile Sorting the Barriers into Respective Groups

System Issue	User Issue	Service Provider Issue	Socio-Culture Factor
No mass vaccination^1^	Vaccine confidence gap^2^	Health Worker Poor Knowledge about dose administration^3^	Children in the lowest wealth quintiles are still the least likely to receive immunizations^4^
No active out-reach activities including notification services and supplementary immunization activities for migrants^5^	vaccinations are delayed beyond recommended ages, alternative schedules are used, or vaccines are totally declined^6^	Insufficient screening of high risk group including migrants^7^	Cultural beliefs and norms among groups from different socioeconomic positions and racial/ethnic minorities, and the impact these differences can have on vaccine confidence.^8^
allocation of insufficient public resources to increase awareness and implement guideline^9^	lack of awareness on HBV in General population and high risk group^10^	Fear of vaccine wastage^11^	Female subjects had better vaccine compliance as compared to males^12^
Coverage National Immunization Program (NIP) vaccines is low^13^	Negative Screening Result So didn’t feel the need of vaccination^14^	Incomplete recording and reporting^15^	Cultural factors may reduce access to health care by women in the postpartum period^16^
Lack of PPP in immunization17	low birth weight baby^18^	Poor stock management^19^	significantly associated with more siblings in household, shorter duration of living in the surveyed areas, lower family income, mother with a job, mother with poor awareness of vaccination, and mother with lower education level.^20^
The mono valent vaccine is not funded so becomes costly^21^	Name of child changes in the first year of life^22^	Poor communication^23^	Low vaccine coverage was associated with poor family wealth status, less than 8 years of maternal schooling and smaller time of residence in the urban area of the city, being related to socioeconomic inequities^24^
Parents wanted vaccination information to be available at a wider variety of locations, including outside health services (low confidence) and in good time before each vaccination appointment^25^	Mobility of families^26^	Insufficient training of HW^27^	Migrants from non-urban areas and children from low income families low coverage^28^
Private health facilities have been shown to lack knowledge on immunization schedule^29^	Lack of opportunity^30^	Incomplete knowledge and awareness among doctors, dentist and nurse/HW^31^	Failure to understand the importance of vaccination Requirement for translation issue of Indian, Bengali, Turkish, or Vietnamese origin^32^
Quality of outreach services^33^	Fear of needles^34^	No home visits to identify unimmunized children^35.^	Coverage with a timely birth dose of HBV vaccine is still low among high risk and rural area^36^
Patent protection remains the major barrier to the production of affordable small-molecule generics^37^	Parent concern on adverse effect^38^	Vaccine storage^39^	Prematurity birth of newborn^40^
No sufficient generic vaccine in market^41^	Mother awareness^42^	Conflicting guideline at hospital^43^	Birth at home^44^
Monetary incentives^45^	Vaccine compliance was lower in health professionals^46^	long delays to vaccine access^47^	Ethnic minority^48^
Logistics are not yet adequate^49^	Caregiver education/Awareness^50^	Vaccine spacing errors^51^	Media report on adverse effect^52^
Poor Hepato Cellular Cancer surveillance^53^	Fear of Side effect^54^	Identification of all high risk pregnancies^55^	Decrease the proportion of women giving birth at health care facilities^56^
Inefficient strategies for tribal and high-risk groups^57^	Parent unwilling^58^	No routinely test for HBsAg during prenatal visit of women who engage in behaviors that put them at high risk for infection^59^	
Low effectiveness of the primary health care system^60^	False Contraindication^61^	Cold chain^62^	
High costs of HBIG^63^		Health education plus 'reminder-type' immunization cards to remind caregivers^64^	
Outreach Inconvenience^65^		Poor communication and negative relationships with health workers^66^	
lack of co-ordination of various level^67^		Maternal HBV DNA levels and detectable HBV DNA in the cord blood^68^	
Lack of official communication^69^		High levels of viremia in mothers^70^	
System Issue	User Issue	Service Provider Issue	Socio-Culture Factor
No mechanism for recording birth dose71		Subject with HBV infection not aware of their infection72	
cost of vaccine73		Private maternity services74	
22	16	22	14
29.70%	21.60%	29.70%	18.91%

1, (Butsashvili et al., 2012; Giles-Vernick et al., 2016b; Larcher et al., 2001b); 2, (Cox et al., 2012b; Fourn et al., 2009b; Ritvo et al., 2003); 3, (Butsashvili et al., 2012; Ko et al., 2017b); 4, (Branco et al., 2014; Wu et al., 2017); 5, (Han et al., 2014); 6, (Bowman et al., 2014; MacDougall et al., 2015b); 7, (Han et al., 2014; Philbin et al., 2012b); 8, (Philbin et al., 2012b); 9, (Akibu et al., 2018; Butsashvili et al., 2012; Harris et al., 2007); 10, (Juon et al., 2009b; Khan & Ross, 2013; Yau et al., 2016b; Zacharias et al., 2015); 11, (Butsashvili et al., 2012; MacDougall et al., 2015b); 12, (Navarro et al., 2014); 13, (Akibu et al., 2018; MacDougall et al., 2015b; Mollema et al., 2012); 14, (Chingle et al., 2017); 15, (Akibu et al., 2018; Branco et al., 2014); 16, (Butsashvili et al., 2012; MacDougall et al., 2015b; Philbin et al., 2012b); 17, (Kabir et al., 2010); 18, (Harris et al., 2007); 19, (Han et al., 2014); 20, (Ganczak et al., 2015; Larcher et al., 2001b; Philbin et al., 2012b; Wu et al., 2017); 21, (Akibu et al., 2018; Ayalew & Horsa, 2017; MacDougall et al., 2015b; Zacharias et al., 2015); 22, (Branco et al., 2014); 23, (Mollema et al., 2012); 24, (Zacharias et al., 2015); 25, (Mollema et al., 2012); 26, (Branco et al., 2014); 27, (Ko et al., 2017b); 28, (Branco et al., 2014; Ganczak et al., 2015); 29, (Giles-Vernick et al., 2016b; Jaquet et al., 2017); 30, (MacDougall et al., 2015b; Said & Jou, 2014c); 31, (Ganczak et al., 2015; Jaquet et al., 2017; Kelling et al., 2016); 32, (Mollema et al., 2012); 33, (Han et al., 2014); 34, (Chingle et al., 2017); 35, (Branco et al., 2014; Wu et al., 2017); 36, (Branco et al., 2014; Harris et al., 2007); 37, (Akibu et al., 2018; Ritvo et al., 2003); 38, (Butsashvili et al., 2012; Ritvo et al., 2003); 39(Harris et al., 2007); 40, (Butsashvili et al., 2012); 41, (Akibu et al., 2018); 42, (Navarro et al., 2014); 43, (Harris et al., 2007); 44, (Wu et al., 2017); 45, (Bowman et al., 2014); 46, (Jaquet et al., 2017); 47, (Butsashvili et al., 2012; Chingle et al., 2017); 48, (Fourn et al., 2009b); 49, (MacDougall et al., 2015b); 50, (Ritvo et al., 2003; Wu et al., 2017); 51, (Kelling et al., 2016); 52, (Mollema et al., 2012); 53, (Yue et al., 2018b); 54, (Butsashvili et al., 2012); 55, (Jaquet et al., 2017); 56, (Wu et al., 2017); 57, (Harris et al., 2007); 58, (Fourn et al., 2009b; Mollema et al., 2012); 59, (Bowman et al., 2014); 60, (Ganczak et al., 2015); 61, (Fourn et al., 2009b); 62, (MacDougall et al., 2015b); 63, (Akibu et al., 2018); 64, (Oyo-Ita et al., 2016); 65, (Chingle et al., 2017; Ganczak et al., 2015); 66, (Butsashvili et al., 2012); 67, (Akibu et al., 2018); 68, (Navabakhsh et al., 2011); 69, (Mollema et al., 2012); 70, (Navabakhsh et al., 2011); 71, (MacDougall et al., 2015b); 72, (Larcher et al., 2001b); 73, (Butsashvili et al., 2012); 74, (Ganczak et al., 2015; Wu et al., 2017).

## Discussion

HBV remains a key challenge despite being prevented by vaccine (Wang et al., 2016). In several trails HB vaccine has been proven to be safe for dispensing among infants, children, adolescents and adults (Advisory Committee on Immunization Practices and Centers for Disease Control and Prevention (CDC), 2011; Azami et al., 2016; Schillie et al., 2013) Still only 30% of HCWs intended to be vaccinated and others didn’t want to get vaccinated as they had apprehension regarding efficacy and safety of vaccines. This reflects the low willingness for vaccination and shows the low knowledge level of HB vaccination (Wang et al., 2016).

This study describes the awareness of vaccine prevention of HBV, the risk perception of HBV infection, barriers of HBV vaccine. It is never possible to eradicate HBV unless adequate person who are at risk of HBV are identified (Goldstein et al., 2002). This study shows a wide variation in target group for HBV ranging from general population to high risk population, which would need service provider with proper knowledge and logistic support.

Vaccination is hard to occur due to variety of barriers like cultural barriers (Philbin et al., 2012a) time constraints (Juon et al., 2009a; Said and Jou, 2014a), and dearth of awareness related to HBV (Park et al., 2013) HBV infection among high risk group and knowledge regarding them (Yue et al., 2018a). Cost of vaccine even acts as a hindrance (Said and Jou, 2014b) as it is seen that at risk for HBV do not have an insurance coverage.

In this study “*Lack of knowledge about Hepatitis B vaccination and having low education among care giver*” is mentioned in more than 33% of the reviewed studies. Followed by “*False belief regarding vaccination and cultural barriers*” by 25% and similarly “*Cost of vaccine and unavailability of the vaccine” mentioned by 25% of reviewed studies. We found* “low awareness among providers and Inadequate knowledge of HBV among health-care workers” which mentioned by 19% (7) of reviewed studies. 

From literature review it is seen that self- efficacy has been lacking in the guidelines during practice (Cox et al., 2012a), language barrier (*Adherence to the Screening Program for HBV Infection in Pregnant Women Delivering in Greece, n.d.*), lack of funding for adult vaccines (Yau et al., 2016a), immigrant women (Papaevangelou et al., 2006), inertia of previous practice (Giles-Vernick et al., 2016a), ethnicity and place of origin being a barrier (Philbin et al., 2012a), mother had delivered elsewhere (Larcher et al., 2001a), patient-related barriers (Leng et al., 2016), vaccination goes against the will of God (Fourn et al., 2009a), misconception that vaccine are meant for children and the perception among healthcare providers, general public (Yau et al., 2016a) and several other external barriers also affect vaccine uptake (Ko et al., 2017a).

This study has identified 74 barriers among all the reviewed papers, further the barriers are grouped as “*System Issue*”, “*User Issue*”, “*Service Provider Issue*” and “*Socio-Culture Factor*”. One-third of the barriers are related to system issues, one-fourth of the barriers were related to caregiver education or awareness, fear of side effect, migration communication and/relation with the caregivers, failure to identify unimmunized children etc., and other barriers were social-cultural issues. 

In order to reduce the barrier, measures like health education, awareness programme, formulate a policy benefiting HBV vaccination is much needed. 

*Future Possibilities*

There are scopes to increase HBV vaccination and screening rates can be done by mass educational programs, awareness among high risk groups for HBV. Such interventions can be considered for increasing the vaccine uptake.

The Healthy People 2020 disease reduction goals has been put forth in United States to achieve control of HBV and thus similar steps could be implemented in other countries after thorough research(Said and Jou, 2014b).

In Conclusion the review reveals limited availability and accessibility to health-facility based immunization, lack of awareness among caregivers, poor communication by the healthcare workers and negative relationships with the beneficiaries, cost of vaccine in private sector, inconvenience time and place of vaccination etc. as the major barriers for vaccine uptake. Barriers varied from country to country. One-fifth barriers were related to service provider issues like poor out-reach, home visits.

However, HBV can be eradicated but several hurdles and obstacles acting as barriers need to be resolved. The difficulties to bridle vaccine coverage are by reducing gaps and increase coverage rates, focusing on high risk targets, treatment of prolong chronically infected persons, minimizing the breach within routine practice and recommendation.

A decadal constant attempt will make HBV infection potential enough to be contemplate for eradication at a global level alike small pox and polio. This needs a vigorous effort to achieve it.

A planned, proper hepatitis B vaccination policy all through the country should be prioritized to have better vaccine coverage. Vaccine coverage should be targeted to at-risk group, individuals unknowingly having chronic HBV infection, who are capable to transmitting the infection. An Improved guideline on vaccination management should be adapted.

Our study has few limitations while interpreting the reviewed data. In addition, this study used pile sorting techniques, which while grouping the barriers may provide at times fairly consistent results between participants, or may vary widely. The findings may not be generalized to all population. Future studies should be triangulated with other study types which elicit recent barriers and facilitators of HBV vaccination.
